# Analysis of Important Volatile Organic Compounds and Genes Produced by Aroma of Pepper Fruit by HS-SPME-GC/MS and RNA Sequencing

**DOI:** 10.3390/plants12122246

**Published:** 2023-06-08

**Authors:** Yinhui Qiu, Yongqing Li, Lidong Wu, Hang Wei, Jianwei Fu, Weiting Chen, Shuting Lin, Sheng Yang, Rui Zhang, Wei Shang, Chengshu Liao, Shaogui Zeng, Ying Luo, Weiwei Cai

**Affiliations:** 1 Sanming Academy of Agricultural Sciences, Sanming 365509, China; qq309686995@163.com (Y.L.); smnkswld@163.com (L.W.); chenwaiting1234@163.com (W.C.); qq1209674734@163.com (S.L.); zhangrui01a@163.com (R.Z.); smshangwei@163.com (W.S.); laoliao868@163.com (C.L.); zsg17350569668@126.com (S.Z.); luoying1966@126.com (Y.L.); 2 Fujian Key Laboratory of Crop Genetic Improvement and Innovative Utilization for Mountain Area, Sanming 365509, China; 3 Institute of Agricultural Quality Standards and Testing Technology, Fujian Academy of Agricultural Sciences, Fuzhou 350002, China; qq383982717@163.com; 4 Key Laboratory of Applied Genetics of Universities in Fujian Province, Fujian Agriculture and Forestry University, Fuzhou 350002, China; fjw9238@163.com (J.F.); 2022019@cau.edu.cn (S.Y.); 5 College of Horticultural Sciences, Zhejiang Agriculture and Forestry University, Hangzhou 350002, China

**Keywords:** *Capsicum annuum*, aroma, volatile organic compounds, fatty acid biosynthesis, terpene synthesis

## Abstract

Pepper is an important condiment, and its aroma affects its commercial value. In this study, transcriptome sequencing and combined headspace solid-phase microextraction and gas chromatography–mass spectrometry (HS-SPME-GC-MS) were used to analyze the differentially expressed genes and volatile organic compounds in spicy and non-spicy pepper fruits. Compared with non-spicy fruits, there were 27 up-regulated volatile organic compounds (VOCs) and 3353 up-regulated genes (Up-DEGs) in spicy fruits. The results of KEGG enrichment analysis of the Up-DEGs combined with differential VOCs analysis showed that fatty acid biosynthesis and terpenoid biosynthesis may be the main metabolic pathways for aroma differences between non-spicy and spicy pepper fruits. The expression levels of the fatty acid biosynthesis-related genes FAD, LOX1, LOX5, HPL, and ADH and the key terpene synthesis gene TPS in spicy pepper fruits were significantly higher than those in non-spicy pepper fruits. The differential expression of these genes may be the reason for the different aroma. The results can provide reference for the development and utilization of high-aroma pepper germplasm resources and the breeding of new varieties.

## 1. Introduction

Pepper is one of the world’s most popular vegetables and spices [1,2]. The *Capsicum* genus comprises at least 32 species, of which 5 (*C. annuum*, *C. chinense*, *C. baccatum*, *C. frutescens*, and *C. pubescens*) are domesticated, while the others are classified as semi-domesticated and wild [3]. Their unique aroma components have an important influence on the flavor of fruits and vegetables and their processed products [4]. *C. annuum* and *C. chinense* are the two most widely cultivated peppers in China. The most obvious difference between them is that *C. chinense* has a volatile irritating smell and a unique spicy flavor. Moreover, most *C. chinense* varieties have a distinctive fragrance and are of high commercial value [5,6]. In *C. annuum* varieties, the contents and types of volatile substances have gradually decreased or even disappeared with fruit development in different periods [7].

Terpenoids, most of which have strong biological activity and aroma, are important raw materials for food, medicine, cosmetics, and spices [8]. Terpenoids are closely related to the production of fruit aroma [9,10,11]. Volatile terpenes mainly include isoprene, monoterpenes, and sesquiterpenes and its derivatives [8]. Terpenoids are the main volatile components in pepper. These terpenoids are not only produced by the metabolism of pepper, but also by the decomposition of macromolecular terpenoids (such as carotenoids) in storage [12]. In addition to terpenoids, pepper also contains small amounts of aldehydes, ketones, alcohols, esters, and other substances, most of which come from the decomposition and esterification of fatty acids [13,14]. Some aldehydes, ketones, and esters have a low flavor threshold and tend to have the characteristic green smell and fruity aroma of pepper. They may also be important flavor components in pepper [13,15].

Volatile organic compounds (VOCs) can be grouped according to their biosynthetic origins as fatty acid derivatives, amino acid derivatives, terpenoids, phenylpropanoids/benzenoids, and species- or genus-specific compounds not involved in those major classes [16,17]. As an important attribute of the sensory quality of pepper, VOCs are one of the important indicators for measuring the commerciality of pepper, which has been paid more and more attention by pepper producers, processing enterprises, and consumers [18]. Because of its high nutritional value, it is necessary to study and evaluate its potential as a functional food and active compound. Like most of the quality traits of the pepper fruit, the variation of volatile substances is the result of the combination of genotype and environment, and it may be jointly regulated by many genes and metabolic networks [19,20,21]. The types and contents of volatile substances vary according to the cultivated species, the fruit’s development stage, and the fruit’s location [22,23]. At present, although there are some studies on the types, the sources, and influencing factors of pepper aroma substances, most of them focus on dried chili and its products, the aroma substances and their relationship with external factors, and most of them concern the expression of their appearance, and there is a lack of in-depth research on the formation mechanism of pepper aroma [24,25].

Combined transcriptome (RNA-Seq) and headspace solid-phase microextraction (HS-SPME) with gas chromatography coupled to mass spectrometry (GC-MS) analysis are widely used to systematically study the formation mechanism of fruit aroma [26,27,28,29]. However, it has not been reported that RNA-Seq and HS-SPME-GC-MS have been used to analyze the formation mechanism of pepper aroma.

In the present study, we investigated two main cultivated pepper species’ (*C. annuum* and *C. chinense*) VOC classification and content, and identified their main odor-contributing volatile components. Moreover, we conducted global expression analysis using RNA-Seq in two main cultivated pepper species (*C. annuum* and *C. chinense*) to identify the key candidate genes controlling pepper aroma that were obtained, and we used qPCR to validate our findings. These results help us to understand the main aroma biosynthesis that affects the fruit quality of capsicum and to improve the fruit quality of pepper in future cultivation.

## 2. Results

### 2.1. Volatile Organic Compounds (VOCs) in Non-Spicy (A) and Spicy (B) Pepper Fruits

**Figure 1:**
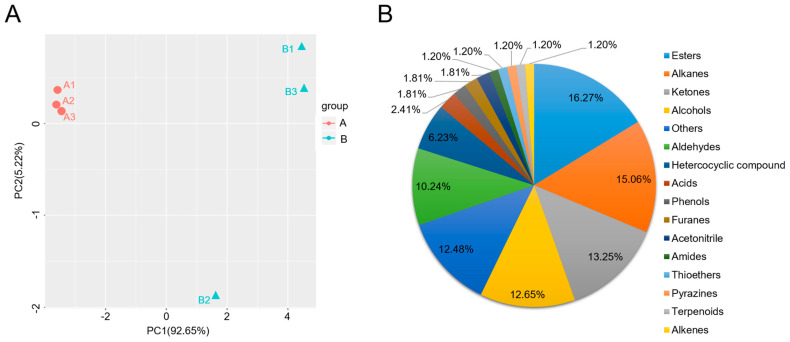
Classification of total 166 volatile organic compounds (VOCs) detected in both non−spicy and spicy pepper fruits. (**A**) Principal component analysis (PCA) of the samples of pepper fruit by HS−SPME−GC/MS at different ripening stages. (**B**) Differential VOCs’ accumulation pattern of pepper fruit.

Through OPLS-DA analysis of the filter metabolites in the orthogonal variables, 88 unrelated to the classification variables and 89 to the non-orthogonal variables and orthogonal variables were analyzed, respectively, to obtain more reliable metabolites among the 90 group differences and the degree of correlation information of the experimental group. The R2Y in OPLS-DA analysis of aroma substances in non-spicy and spicy pepper fruits was 1, indicating that the more stable and reliable the model was, the more successfully the model could be used to screen differential metabolites. Q2Y = 0.944 > 0.9, indicating that this model is an excellent model that can distinguish correct sample groups by metabolite expression (Figure 1A). The analysis of PCA (Principal Component Analysis) results showed that the three biological repeats of non-spicy (A) *C. annuum* were in the same category, and the three biological repeats of spicy (B) *C. chinense* were in the same category, with good repeatability (Figure 1A). A total of 166 volatile compounds was detected in both the non-spicy and spicy capsicum fruits, including 27 Esters (16%), 25 Alkanes (15%), 22 Ketones (16%), 21 Alcohols (13%), 20 Others (12%), 17 Aldehydes (10%), and 11 Heterocyclic compounds (7%), 4 Acids (3%), 3 Phenols (2%), 3 Furans (2%), 3 Cyanides (2%), 2 Amides (1%), 2 Thioethers (1%), 2 Pyrazines (1%), 2 Terpenoids (1%), and 2 Alkenes (1%) (Figure 1B). In addition, the average volatile matter content of spicy pepper fruit (B) was 39.79% higher than that of non-spicy pepper fruit (A).

### 2.2. Differential VOCs in the Non-Spicy (A) and Spicy (B) Pepper Fruits

Based on the quantitative analysis of aroma substances of non-spicy and spicy pepper fruits, 37 different volatile components were found. According to the structural characteristics of the 37 different volatile metabolites, they were further divided into fatty derivatives such as butanal, 3-methyl-, hexanal, 2-Pentenal, (E)-, 2-Hexenal, (E)-, octanal, nonanal, etc., aromatic derivatives such as mequinol, nitrogen and oxygen heterocyclic compounds such as furan, 3-methyl-,Furan, 2-ethyl-,Furan, 2-pentyl-,2(4H)-Benzofuranone, and 5,6,7,7a-tetrahydro-4,4,7a-trimethyl-, (R), and terpenoid derivatives such as 1-Cyclohexene-1-carboxaldehyde, 2,6,6-trimethyl-,Hydroxypivalic acid.

There were 30 fatty derivatives in the differentially volatile components, and there were 22 fatty derivatives. The content of spicy pepper fruit (B) was significantly higher than that of non-spicy pepper fruit (A), and the content of 1-Hexanol, 2-Hexen-1-ol, (E)-, 2-Hexenal, (E)-, 1-Penten-3-ol, Hexanal and other fatty derivatives was higher. Moreover, the content in non-spicy pepper fruit (A) had the largest difference (Table 1). In addition, the content of 1-Cyclohexene-1-carboxaldehyde,2,6,6-trimethyl-, a terpenoid derivative in spicy pepper fruit (B) was significantly higher than that contained in non-spicy pepper fruit (A). These results suggest that fatty derivatives may be an important reason for the difference in aroma between non-spicy and spicy pepper fruits.

### 2.3. Overview of Transcriptomic Data for 17-03 and H1023 Non-Spicy (A) and Spicy (B) Pepper Fruits

An Illumina sequencing platform was used for high-throughput sequencing analysis of cDNA from pepper fruit samples. After sequencing quality control of the original data, the redundant sequences and low-quality reads were removed, generating 28.87 Gb of clean data. At least 2.21 Gb of clean data were generated for each sample, with a minimum of 92.87% of the clean data achieving a quality score of Q30 (Table 2). The clean reads of each sample were mapped to the specified reference genome. The mapping ratio ranged from 89.67% to 94.55% (Table 3).

The mapping ratio refers to the percentage of mapped reads in clean reads, which indicates the utilization of RNA data. Besides the influence of sequencing data quality, the mapping ratio is also affected by the quality of reference genome assembly and the biological classification relation between the sequenced sample and reference subspecies. The mapping ratio is an important parameter for examining whether the reference genome is suitable for following bioinformatic analysis.

### 2.4. Identification of Differentially Expressed Genes (DEGs)

For experiments with biological replicates, differential expression analysis is processed by DESeq2 [30]. For projects without biological replicates, edgeR [31] is applied. The criteria for differentially expressed genes were set as Fold Change (FC) ≥ 2 and FDR < 0.01. Fold change (FC) refers to the ratio of gene expression in two samples. False Discovery Rate (FDR) refers to adjusted *p*-value, which is used to measure the significance of difference.

**Figure 2:**
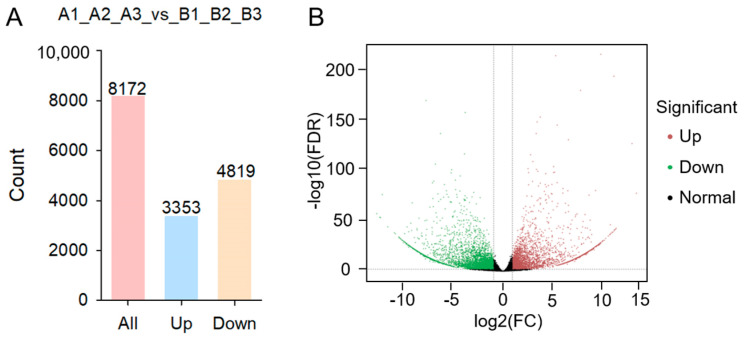
Expression analysis of differentially expressed genes (DEGs) in non−spicy vs. spicy pepper fruits. (**A**) Numbers of up− and down-regulated DEGs in non-spicy vs. spicy pepper fruits. (**B**) Volcano plot of up− and down-regulated DEGs in non−spicy vs. spicy pepper fruits.

The FPKM algorithm was used for differential expression analysis among the samples, and the results showed that there was a total of 8172 differentially expressed genes between the non-spicy (A) and spicy (B) pepper fruits, of which 3353 genes of spicy pepper fruit (B) were up-regulated and 4819 genes of spicy pepper fruit (B) were down-regulated relative to non-spicy pepper fruit (A) (Figure 2).

### 2.5. KEGG Functional Annotations of DEGs

**Figure 3:**
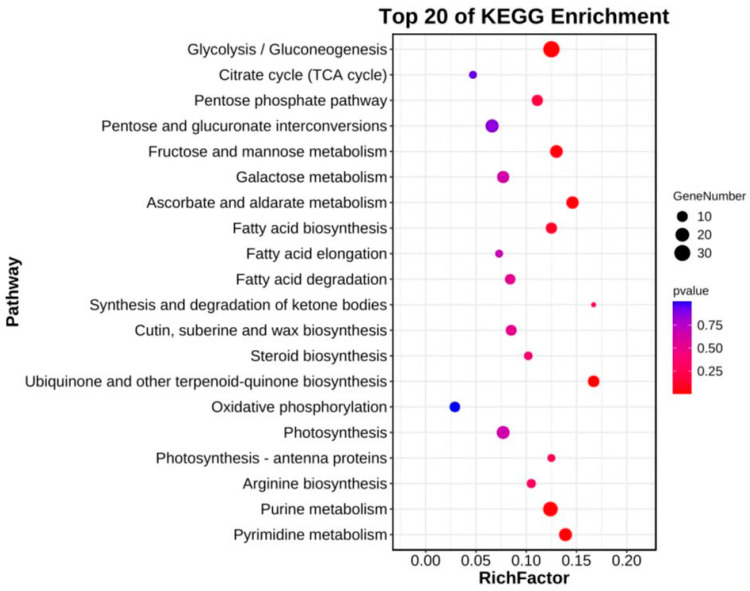
The top 20 enriched KEGG pathways for DEGs of up-regulated genes in spicy (B) vs. non-spicy (A) pepper fruits.

In this session, we examined if the pathways are over-presented with DEGs. Enrichment factors and Fisher’s test were applied in the determination of enrichment degree and significancy of the pathway. The enrichment of DEGs in KEGG pathways is shown in the figures below. The top 20 different expressed up-regulated gene-enriched pathways (with smallest Q-value) are shown (Figure 3).

The KEGG metabolic pathways that significantly up-regulated gene enrichment of spicy (B) and non-spicy (A) pepper fruits mainly included fatty acid biosynthesis, ubiquinone and other kinds of terpenoid-quinone biosynthesis, glycolysis/gluconeogenesis, pentose phosphate pathway, fructose and mannose metabolism, citrate cycle (TCA cycle), arginine biosynthesis, purine metabolism, pyrimidine metabolism, and other metabolic pathways. On the strength of the analysis results of the VOCs in the non-spicy (A) and spicy (B) pepper fruits, the biosynthesis of fatty acid biosynthesis, fatty acid biosynthesis and ubiquinone and other kinds of terpenoid-quinone biosynthesis may be the main metabolic pathways of aroma differences between non-spicy and spicy pepper fruits.

### 2.6. Analysis of DEGs Related to Fatty Acid and Terpene Biosynthesis

**Figure 4:**
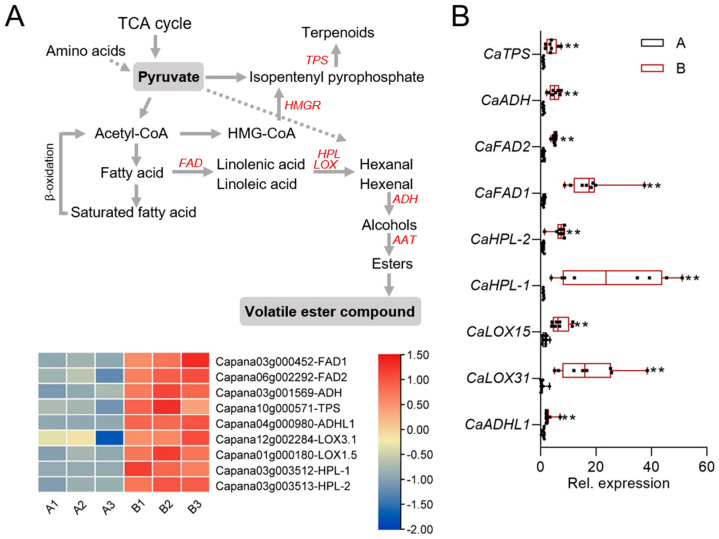
DEGs of fatty acid and terpene biosynthesis. (**A**) The fragments per kilobase million (FPKM) of DEGs in fatty acid and terpene biosynthesis. (**B**) qRT−PCR-based validation of DEGs in fatty acid and terpene biosynthesis. Data represent of eight replicates. *CaActin* was used as an internal control, and asterisks above the bars indicate significant differences between means (*p* < 0.01), as calculated with Fisher’s protected *t* test. The center line represents the median value and the boundaries indicate the 25th percentile (upper) and the 75th percentile (lower). Whiskers extend to the largest and smallest value.

The results of up-regulated differential metabolites and differential gene KEGG enrichment in non-spicy (A) and spicy (B) pepper fruits indicated that the differential expression of genes related to fatty acid biosynthesis, fatty acid elongation, and ubiquinone and other terpenoid-quinone biosynthesis pathways may be the key reason for the aroma differences between two kinds of pepper fruits. Through transcriptome data screening, the key genes of the fatty acid biosynthesis pathway in spicy pepper fruit (B) were found to be FAD (Capana03g000452, Capana06g002292), LOX (Capana12g002284, Capana01g000180), HPL (Capana03g003512, Capana03g003513), and ADH (Capana04g000980). The above key gene expressions in spicy pepper fruit (B) were significantly higher than those in non-spicy pepper fruit (A). In addition, TPS (Capana10g000571), a key gene for ubiquinone and other terpenoid-quinone biosynthesis pathways, was significantly up-regulated in spicy pepper fruit (B) (Figure 4A). In order to confirm the accuracy of the transcriptome sequencing results, qRT-PCR was performed to verify the differentially expressed genes above, and the results showed that the expression levels of the above genes in spicy pepper fruit (B) were significantly higher than those in non-spicy pepper fruit (A) (Figure 4B).

## 3. Discussion

Aroma is an important feature of pepper fruit flavor and quality, which is closely related to VOCs in pepper fruit [32,33]. In this study, headspace solid-phase microextraction and gas chromatography–mass spectrometry (HS-SPME-GC-MS) combined with transcriptome were used to reveal the differential gene expression of aroma components and related metabolic pathways in non-spicy and spicy pepper fruit. It is not only helpful to understand the main VOCs formed by different aroma, but also to further explore the main metabolic pathways and potential key genes forming aroma differences. The purpose of this study is to provide a theoretical basis for future molecular breeding of pepper aroma.

### 3.1. The Contents of Fatty Acids and Terpenes Affect the Aroma Production of Capsicum Fruit

The main VOCs in pepper fruits are esters and terpenes. Other trace compounds include lipoxygenase derivatives, nitrogen–sulfur compounds, phenolic derivatives, norcarotene, carbonyl groups, alcohols, and other hydrocarbons [5,34]. In this study, it was found that there were 166 kinds of VOCs in non-spicy (A) and spicy (B) pepper fruits, of which esters accounted for the largest proportion, which were the main VOCs in the two kinds of pepper fruits. The average volatile content of spicy pepper fruit (B) was significantly higher than that of non-spicy pepper fruit (A). According to this result, we think that the content of VOCs determines the formation of the aroma of pepper fruit, not the type of VOCs.

According to the structural characteristics, VOCs can be divided into fatty derivatives, terpene derivatives, aromatic derivatives, and nitrogen-containing and oxygen-containing heterocyclic compounds [35]. The content of 27 VOCs in spicy pepper fruit (B) was significantly higher than that in non-spicy pepper fruit (A), among which fat derivatives accounted for the highest proportion. The contents of n-hexanol, trans-2-hexenol, 2-hexenal-2, 1-pentene-3-ol, n-hexanal, nonanal, geranyl acetone, and other fatty derivatives in spicy pepper fruit are quite different from those in non-spicy pepper fruit, which may be an important reason for the aroma difference between spicy pepper fruit and non-spicy pepper fruit (A) with strong green aroma and flower and fruit aroma. In previous studies, sniffing analysis showed that the odors produced by peppers were green leaves, cucumbers, irritants, or herbs [36]. During the ripening process of pepper, fatty derivatives are accumulated, and they can synthesize VOCs using fat and linolenic acid as precursors, thus giving pepper fruit a green aroma [17]. In this study, spicy pepper fruit (B) contained higher levels of trans-2-hexenol and 2-hexenal, which are fatty derivatives.

Due to the odor threshold, not all VOCs could significantly affect the formation of pepper aroma. Previous studies have found that n-hexanal, 2-hexenal, and nonanal can significantly promote the formation of pepper aroma [37], and these substances also have a high content in spicy pepper fruits (B). Floral and fruity aroma is another important feature of pepper aroma; β-ring citral, n-hexanol, and 1-penten-3-ol have sweet fruity aroma; nonanal has a citrus and orange-like odor; and geranyl acetone has a woody floral scent [38]. In spicy pepper fruit (B), the content of β-ring citral, n-hexanol, and 1-penten-3-ol is higher, which makes it produce a more intense fruit aroma. However, the characteristic flavor of pepper fruit is related to the type and combination ratio of VOCs, and the proportion of the unique flavor of spicy pepper fruit (B) needs further study.

### 3.2. The Key DEGs in the Aroma of Spicy Pepper Fruits

In this study, transcriptome sequencing was used to explore the main metabolic pathways and potential key genes responsible for the aroma difference between non-spicy (A) and spicy (B) pepper fruits. Through KEGG functional enrichment of up-regulated expressed genes in spicy pepper fruits (B), it was found that it contains multiple metabolic pathways related to the formation of aroma differences: fatty acid biosynthesis and extension related to fatty derivatives, biosynthesis of ubiquinone and other terpenoids related to terpenoid biosynthesis, glycolysis/gluconeogenesis, pentose phosphate, fructose and mannose metabolism related to the biosynthesis of aroma substances with monosaccharides and glycosides as precursors, and the metabolism of arginine biosynthesis related to the synthesis of aroma substances from amino acids as precursors (Figure 3). Previous studies have shown that C6 and C9 aldehydes are the main contributors to the green aroma and are involved in fatty acid metabolism [32]. Unsaturated fatty acids are oxidized to form 9-hydroperoxy and 13-hydroperoxy intermediates, which are further metabolized by two lipoxygenase (LOX) pathway branches to produce VOCs such as n-hexanal, nonanal, and 2-hexenal, which are high in spicy pepper fruit (B) [39]. Terpenoids are one of the important components of fruit aroma, and the content of β-cyclic citral B is high in spicy pepper fruit (B). Therefore, the biosynthesis pathway of terpenes such as ubiquinone may also be an important factor in the formation of aroma differences [16,40]. In addition, the synthesis of aroma substances also involves biosynthesis with amino acids as precursors. Therefore, it is speculated that the biosynthesis of arginine may also be related to the formation of aroma differences.

Combined with the results of differential volatile component analysis and differential gene metabolic pathway analysis of non-spicy (A) and spicy (B) pepper fruits, the results showed that fatty acid biosynthesis and biosynthesis of terpenoids such as elongation and ubiquinone may be the main metabolic pathway for aroma differences between non-spicy (A) and spicy (B) pepper fruits. Fatty acids form linoleic acid and linolenic acid under the action of fatty acid desaturase (FAD); LOX recognizes and binds linoleic acid and linolenic acid, deoxidizes the substrate, and then decomposes and metabolizes peroxisome lyase (HPL) to produce oxygen-containing acids and volatile aldehydes; and volatile aldehydes can be converted into alcohols by alcohol dehydrogenase (ADH) [41,42,43]. It was found that the expression levels of FAD, LOX1, LOX5, HPL, and ADH genes in spicy pepper fruit (B) were significantly higher than those in non-spicy pepper fruit (A). These genes were highly related to the synthesis of fat derivatives. In addition, terpene synthase (TPS) is the key enzyme of terpene biosynthesis pathway, and the expression level of TPS in pepper fruit (B) is significantly higher than that in non-spicy pepper fruit (A). The high expression of these genes may be the reason why the synthesis of fat derivatives and terpenes in spicy pepper fruit (B) is higher than that in non-spicy pepper fruit (A) and form the aroma of pepper fruit. However, in most cases, the use of pepper fruit aroma is in ripe fruit. However, in future research, we should further consider using fruits at different development stages as research materials for analysis because this is more conducive to understanding the differences of important metabolites and gene expression in different stages of pepper fruit aroma formation.

Conclusions: We analyzed the important VOCs and key genes of aroma formation in spicy pepper fruit (B) by the combination of HS-SPME-GC/MS and RNA-seq. It was found that the difference of fat derivatives and terpene synthesis was an important reason for the aroma of pepper fruit.

## 4. Materials and Methods

### 4.1. Plant Materials and Growth Conditions

**Figure 5:**
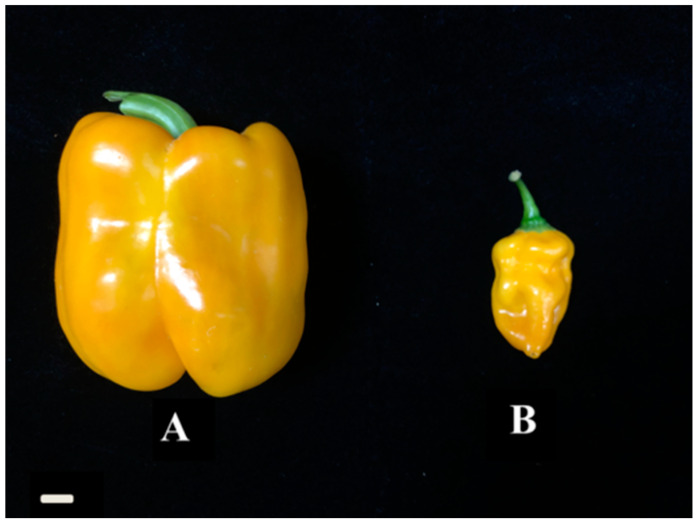
Two pepper inbred lines with different aroma. (**A**) *C. annuum* (not spicy). (**B**) *C. chinense* (spicy).

A: *C. annuum* high generation inbred line (fruit yellow, not spicy) and B: *C. chinense* (fruit yellow, spicy) (Figure 5). The seeds of the two pepper varieties came from Sanming Academy of Agricultural Sciences and were planted in the high heat pepper artificial climate chamber of the Vegetable Research Institute of Sanming Academy of Agricultural Sciences in the spring of 2021. On 15 January, the seedlings were sown and cultivated, and, on 25 March, the pepper was planted in the artificial climate chamber with high spiciness. Each material was planted with 20 POTS, and the conventional cultivation and management were carried out. After the fruits were ripe, 6 fruits of A: *C. annuum* and B: *C. chinense* were selected and mixed into a biological replicate: a total of 3 groups of biological replicates.

### 4.2. Sample Preparation and GC-MS Analysis

Take a 100 mg sample into the 20 mL headspace bottle, add 10 μL of 2-Octanol (10 mg/L stock in dH_2_O) as internal standard, and set the extraction time at 30 min. All samples were analyzed by a gas chromatography system coupled with a spectrometer (GC-MS) in an SPME cycle of a PAL rail system. The type of 57299-USPME (divinylstyrene/carbon box/polydimethylsiloxane DVB/CAR/PDMS) fiber was used. The incubate temperature was 60 °C; the preheat time was 15 min; the incubate time was 30 min; and the desorption time was 4 min. GC-MS analysis was performed using an Agilent 7890 gas chromatography system (The system utilized a DB-Wax, injected in Splitless Mode) coupled with a 5977B mass spectrometer. Helium was used as the carrier gas, the front inlet purge flow was 3 mL min^−1^, and the gas flow rate through the column was 1 mL min^−1^. The initial temperature was kept at 40 °C for 4 min, then raised to 245 °C at a rate of 5 °C min^−1^ and kept there for 5 min. The injection, transfer line, ion source, and quad temperatures were 250, 250, 230, and 150 °C, respectively. The energy was −70 eV in electron impact mode. The mass spectrometry data were acquired in scan mode with the *m*/*z* range of 20–400 and a solvent delay of 0 min. Chroma TOF 4.3X software of LECO Corporation and a Nist database were used for raw peaks’ exacting, the data baselines’ filtering and calibration of the baseline, peak alignment, deconvolution analysis, peak identification, integration, and spectrum match of the peak area.

### 4.3. RNA Extraction and RNA-Seq Analysis

RNA extraction, transcriptome sequencing, and data analysis were commissioned by Beijing Baimaike Cloud Technology Co., LTD. The total RNA was extracted by the Trizol method (Invitrogen). RNA concentration and purity were measured by NanoDrop 2000 (Thermo Fisher Scientific, Wilmington, DE, USA). RNA integrity was assessed using the RNA Nano 6000 test kit from the Agilent Bioanalyzer 2100 system (Agilent Technologies, Santa Clara, CA, USA). Two cDNA libraries of the *capsicum* non-spicy group (A1, A2, A3) and spicy pepper group (B1, B2, B3) were constructed and sequenced by the Illumina HiSeq platform after quality detection. With the capsicum genome database (URL: http://peppersequence.genomics.cn/ accessed on 19 January 2014) as the reference sequence, annotation high quality clean reads were obtained. The Benjamini–Hochberg method was adopted to calibrate the hypothesis testing probability (*p* value) by multiple hypothesis testing and to obtain the False Discovery Rate, FDR. EdgeR was used for differential expression analysis of the two samples. FDR < 0.01 and Fold Change ≥ 2 were set as thresholds for significant differential expression. The differential expression, functional annotation, and functional enrichment of the genes in the non-spicy and spicy samples were analyzed.

### 4.4. Quantitative Real-Time RT-PCR

qRT-PCR assay was performed following the method of a previous study [44]. The SYBR Premix Ex Taq II system of TaKaRa in Japan was used to measure relative expression levels at the transcription level of specific genes. The total RNA of *Capsicum* was extracted by a Trizol kit (Invitrogen, 15596026). The cDNA obtained by reverse transcription was used as the reaction template, and specific primers were performed according to aroma-related candidate genes. The reaction system (10 μL): cDNA template 1 μL; specific primer (Table 4) 0.3 μL; 2×SYBR 5 μL; ddH_2_O 3.4 μL. Reaction procedure: 94 °C 5 s; 94 °C 30 s; 60 °C 34 s; each group involved 8 biological replicates. The transcription expression levels of CaACTIN (GQ339766) or 18 s rRNA (EF564281) were used as internal parameters to standardize the relative transcription expression levels of the candidate genes to be tested relative to the control group.

## Figures and Tables

**Table 1:** The details of 37 VOCs’ significant difference in two pepper fruits.

NO.	Names	CAS	R.T. (min)	Relative Amount		Up and Down
				A	B	
Fatty derivatives						
1	Butanal, 3-methyl-	590-86-3	3.361	0.0285	0.0052	down
2	Hexanal	66-25-1	7.096	0.0761	2.5828	up
3	2-Pentenal, (E)-	1576-87-0	8.422	0.0059	0.1034	up
4	2-Hexenal, (E)-	6728-26-3	11.088	0.1170	6.2553	up
5	Octanal	124-13-0	13.206	0.0017	0.0109	up
6	Nonanal	124-19-6	16.117	0.0059	0.0163	up
7	5-Ethylcyclopent-1-enecarboxaldehyde		16.645	0.0032	0.0194	up
8	2-Dodecenal, (E)-	20407-84-5	19.78	0.0012	0.0179	up
9	2,4-Di-tert-butylphenol	96-76-4	35.852	0.0257	0.0041	down
10	Acetone	67-64-1	2.345	0.2485	0.0158	down
11	3-Octanone	106-68-3	12.191	0.0037	0.0318	up
12	3-Hepten-2-one	1119-44-4	13.525	0.0061	0.0276	up
13	3-Octanone, 2-methyl-	923-28-4	14.288	0.0351	1.1169	up
14	5-Hepten-2-one, 6-methyl-	110-93-0	14.578	0.0117	0.1538	up
15	2-Cyclohexen-1-one, 3,4,4-trimethyl-	17299-41-1	17.506	0.0028	0.0418	up
16	5,9-Undecadien-2-one, 6,10-dimethyl-, (E)-	3796-70-1	27.131	0.0031	0.0399	up
17	3-Buten-2-one, 4-(2,2,6-trimethyl-7-oxabicyclo[4.1.0]hept-1-yl)-	23267-57-4	29.89	0.0065	0.1235	up
18	1H-Pyrrole-2,5-dione, 3-ethyl-4-methyl-	20189-42-8	34.977	0.0007	0.0029	up
19	1-Penten-3-ol	616-25-1	9.706	0.0217	1.0939	up
20	1-Pentanol	71-41-0	12.326	0.0152	0.1110	up
21	1-Hexanol	111-27-3	15.257	0.0156	1.3680	up
22	2-Hexen-1-ol, (E)-	928-95-0	16.629	0.0055	0.4177	up
23	1-Octen-3-ol	3391-86-4	17.783	0.0019	0.0223	up
24	4,4,6-Trimethyl-cyclohex-2-en-1-ol	21592-95-0	31.422	0.0009	0.0032	up
25	Methyl 2-ethyldecanoate		32.694	0.0073	0.0019	down
26	2-Propanol, 1-chloro-, phosphate (3:1)	13674-84-5	42.542	0.0003	0.0002	down
27	Methyl salicylate	119-36-8	25.255	0.0038	0.0414	up
28	Cyclohexen-1-carbonitrile	1855-63-6	20.345	0.0074	0.0027	down
29	Hexanoic acid, 2-ethyl-	149-57-5	29.281	0.1168	0.0340	down
30	Octanoic acid	124-07-2	31.511	0.0209	0.0049	down
Aromatic derivatives						
31	Mequinol	150-76-5	27.082	0.0054	0.0306	up
Nitrogen and oxygen heterocyclic compounds						
32	Furan, 3-methyl-	930-27-8	3.061	0.0139	0.0028	down
33	Furan, 2-ethyl-	3208-16-0	3.982	0.0030	0.0240	up
34	Furan, 2-pentyl-	3777-69-3	11.485	0.0117	0.0578	up
35	2(4H)-Benzofuranone,5,6,7,7a-tetrahydro-4,4,7a-trimethyl-, (R)-	17092-92-1	36.24	0.0018	0.0232	up
Terpenoid derivatives						
36	1-Cyclohexene-1-carboxaldehyde,2,6,6-trimethyl-	432-25-7	21.822	0.0011	0.0309	up
37	Hydroxypivalic acid	4835-90-9	32.258	0.0120	0.0019	down

A: *C. annuum* (non-spicy). B: *C. chinense* (spicy).

**Table 2:** Overview of read counts of all samples of non-spicy (A) and spicy (B) pepper fruits.

Samples	Clean Reads	GbClean Bases	GC Content	%≥Q30
A1	16,205,787	4,808,073,634	42.55%	93.12%
A2	21,019,032	6,251,941,422	42.36%	93.22%
A3	23,177,163	6,915,966,400	42.27%	93.54%
B1	17,803,462	5,305,305,850	42.54%	93.37%
B2	7,404,704	2,209,849,414	42.48%	92.87%
B3	11,323,565	3,377,464,642	42.70%	93.53%

**Table 3:** Overview of all samples read counts mapping to reference genome.

Samples	Total Reads	Mapped Reads	Uniq Mapped Reads	Multiple Map Reads	Reads Map to ‘+’	Reads Map to ‘−’
A1	32,411,574	30,153,480 (93.03%)	29,180,709 (90.03%)	972,771 (3.00%)	14,970,003 (46.19%)	15,006,995 (46.30%)
A2	42,038,064	39,748,585 (94.55%)	38,393,724 (91.33%)	1,354,861 (3.22%)	19,700,905 (46.86%)	19,778,992 (47.05%)
A3	46,354,326	41,728,496 (90.02%)	40,347,864 (87.04%)	1,380,632 (2.98%)	20,723,470 (44.71%)	20,785,507 (44.84%)
B1	35,606,924	32,174,009 (90.36%)	31,015,903 (87.11%)	1,158,106 (3.25%)	15,969,352 (44.85%)	16,009,903 (44.96%)
B2	14,809,408	13,344,748 (90.11%)	12,922,962 (87.26%)	421,786 (2.85%)	6,635,327 (44.80%)	6,652,940 (44.92%)
B3	22,647,130	20,308,455 (89.67%)	19,647,834 (86.76%)	660,621 (2.92%)	10,092,548 (44.56%)	10,120,926 (44.69%)

**Table 4:** Primers used in quantitative real-time RT-PCR.

Gene Name	Gnen ID	Primer F:	Primer R:
FAD1	Capana03g000452	CCTGTAGCACTTGCAGCTCT	TCTCCAAATGCAAGCAACGC
FAD2	Capana06g002292	CTACCCAAAGCCCAGACCAG	ACTAACCCTCAATGCCCAGC
ADH	Capana03g001569	TGGCCAGTGTGTGCATACAT	ACTGCATCTGAAGGAAGGCC
ADHL1	Capana04g000980	CAAGGGATGGAAGCAGCAGA	GCAACTTTCCATGCTGCTCC
TPS	Capana10g000571	ACCTCACGTAGCCAAACGAG	AAGGGCGTCAACTAAGGCAA
LOX3.1	Capana12g002284	TGCGAAGTGAAGTTAGCCGT	GTTGCTTCCCTCCTCAAGCT
LOX1.5	Capana01g000180	TGCAAACGCGTGAAGAACTG	ATAGTCAGCGGTACCAGGGT
HPL-1	Capana03g003512	TTAGGGCCACTTTGGGATCG	TTGACATCCAGTACCGCCAC
HPL-2	Capana03g003513	CGCCTATCTTGATGCATGGC	AGTCTGTTTGTGCCCTCGTT
Actin	GQ339766	CCTCTCAACCCTAAGGCCAACA	ACGTCCAGCAAGATCCAAACGAA
18SR	EF564281	CCGGTCCGCCTATGGTGTGCACCGG	GCAGTTGTTCGTCTTTCATAAATC

## Data Availability

Data are contained within the article.
